# Models of Cultural Niche Construction with Selection and Assortative Mating

**DOI:** 10.1371/journal.pone.0042744

**Published:** 2012-08-14

**Authors:** Nicole Creanza, Laurel Fogarty, Marcus W. Feldman

**Affiliations:** 1 Department of Biological Sciences, Stanford University, Stanford, California, United States of America; 2 Center for Social Learning and Cognitive Evolution, School of Biology, University of St Andrews, St. Andrews, Fife, United Kingdom; University of Oxford, Viet Nam

## Abstract

Niche construction is a process through which organisms modify their environment and, as a result, alter the selection pressures on themselves and other species. In cultural niche construction, one or more cultural traits can influence the evolution of other cultural or biological traits by affecting the social environment in which the latter traits may evolve. Cultural niche construction may include either gene-culture or culture-culture interactions. Here we develop a model of this process and suggest some applications of this model. We examine the interactions between cultural transmission, selection, and assorting, paying particular attention to the complexities that arise when selection and assorting are both present, in which case stable polymorphisms of all cultural phenotypes are possible. We compare our model to a recent model for the joint evolution of religion and fertility and discuss other potential applications of cultural niche construction theory, including the evolution and maintenance of large-scale human conflict and the relationship between sex ratio bias and marriage customs. The evolutionary framework we introduce begins to address complexities that arise in the quantitative analysis of multiple interacting cultural traits.

## Introduction

Niche construction has recently received attention as an important evolutionary process by which organisms alter the evolutionary pressures on themselves and organisms that share their ecological niche [1]–[7]. Niche construction has usually been considered in an ecological context, and typical examples include the aeration of soil by earthworms or the building of dams by generations of beavers [8], [9]. These environmental changes are mediated by individual organisms and become part of the evolutionary niche into which their offspring (and those of other species) are born [10]. In this way, organisms inherit and develop in an ecological niche altered from previous generations.

Humans have collectively engaged in millennia of niche construction on a spectacular scale, often changing their natural environment beyond recognition and almost certainly altering the course of their own evolution as a result [7]. Humans are also unique in the extent and complexity of their cultural learning, and recent theoretical and empirical work suggests that ‘cultural niche construction,’ where one set of human cultural practices contributes to the evolutionary forces acting on genetic traits or a second set of culturally transmitted traits, can be a powerful force explaining human evolution and behavior [11]–[13], [7]. Similar ideas have been discussed in explorations of both gene-culture coevolution [14] and dual-inheritance theory [15], [16]. Here we follow the gene-culture and culture-culture frameworks proposed by Odling-Smee et al. [4] and Ihara and Feldman [12] in formulating a general model capable of accounting for both.

Culturally transmitted behaviors have been important in human evolution, and humans can also affect aspects of their evolutionary trajectories by influencing their cultural environment (e.g. by farming, migrating, or living in large groups). For example, the advent of dairy farming and animal domestication led, in Europe, to an increase in the frequency of the allele for lactase persistence, allowing more individuals to benefit from drinking milk into adulthood [17]–[19]. Animal domestication also changed aspects of the human immune system as humans came into contact with a variety of new animal pathogens [20]. In this way, the human-constructed cultural niche may affect the evolutionary trajectory of genes; this is one form of niche construction first studied quantitatively by Feldman and Cavalli-Sforza [15]. However, it is also possible that one aspect of a culture or one set of culturally transmitted traits forms a cultural niche that affects either the transmission, persistence, or reproductive contributions of other cultural traits. The resulting joint evolutionary dynamics are characterized by feedback between the different sets of cultural entities. For example, Lipatov et al. [21] describe a model that focuses on traditional Chinese marriage beliefs, which interact with the economic index of a population to influence marriage practices. This concept is sometimes called ‘context dependence’ in the social sciences, and it has received little attention from a quantitative evolutionary point of view.

**Figure 1:**
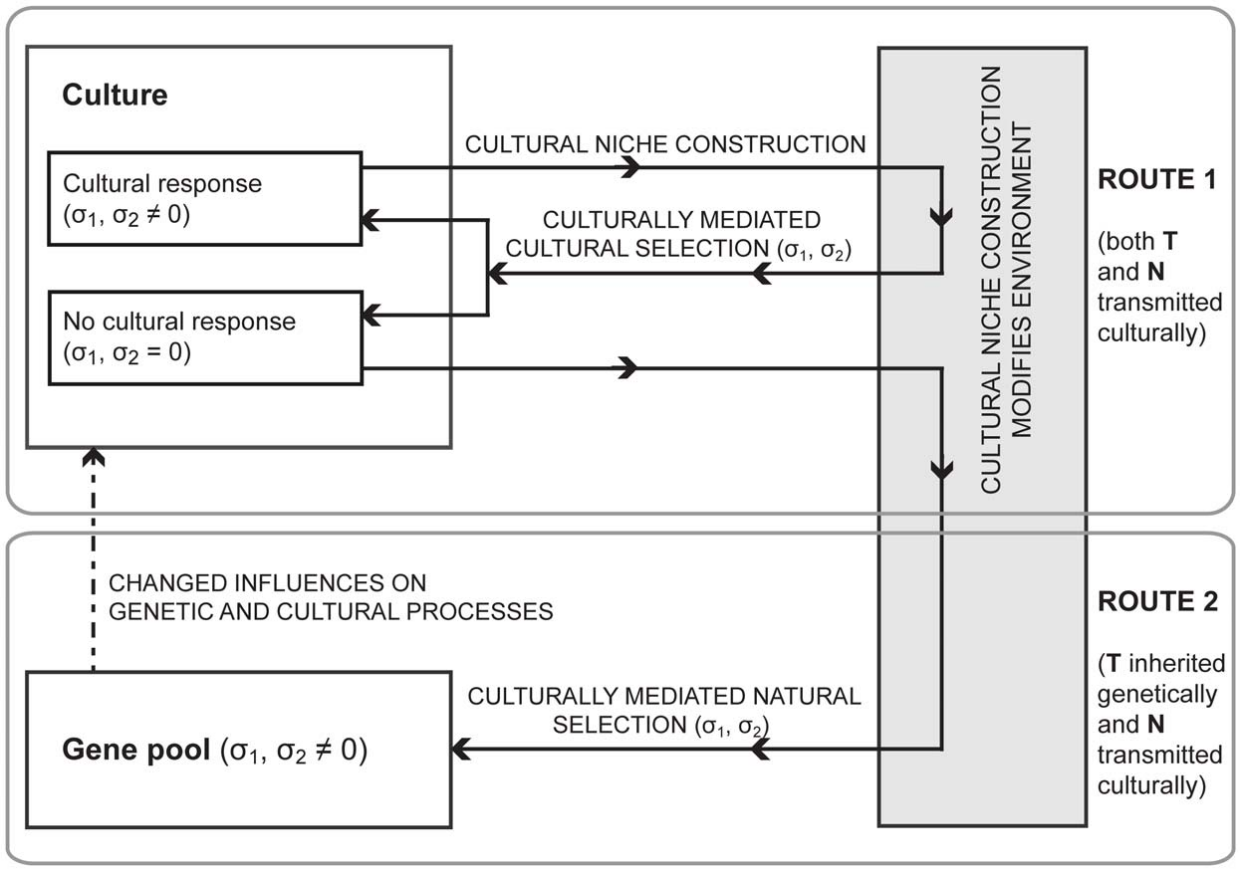
Schematic of cultural niche construction. Cultural niche construction results in environmental variation, which may produce two distinct forms of feedback. Route 1: a cultural trait modifies selection pressures, which can induce further cultural change. Route 2: gene-culture coevolution, where a cultural trait changes selection pressures, causes population level genetic changes in response. Evolutionary outcomes from both route 1 and route 2 depend on the frequency of **T** (cultural or genetic) and **N** (cultural) traits in the population and the selection pressures they generate, here represented by *σ_i_*. Modified from Odling-Smee, Laland, and Feldman (2003).

Here we describe a model of cultural niche construction that formalizes a wide range of evolutionary interactions, including gene-culture interactions, in which a cultural trait can alter selection pressures on a genetic trait or vice versa, and culture-culture interactions, in which a cultural trait alters the evolutionary forces acting on another cultural trait. Our model can represent either type of interaction depending on the rules of transmission, mating, and selection, which generate feedback between one trait and the other (Figure 1). For example, the extent of assortative mating for one trait may influence the evolutionary dynamics of another. Applications of our model include the interaction between religious beliefs and fertility (e.g. [22]), the cultural evolution of large-scale conflict (e.g. [23]), level of education and attitudes towards fertility control (e.g. [12]), male-biased sex preference and marriage practices in Asia (e.g. [21]), or the possible interaction between marriage customs and other cultural beliefs (e.g. [24]). We model two vertically transmitted traits, where each could be considered either genetically or culturally transmitted, and horizontal transmission is incorporated as cultural mutation, where an individual's traits may diverge from those of its parents. Note that although the model could accommodate two genetic traits, here we focus our analysis on cases where at least one trait is culturally transmitted. We also incorporate assorting, an individual's tendency to choose a mate carrying the same trait (either cultural or genetic) as itself, and selection, which allows the relative fitness of the phenotypes to differ. This enables us, for example, to investigate the interaction between assortative mating and any direct selective advantages or disadvantages the traits might bestow. We present a framework that accommodates two interacting cultural traits, which can influence the evolutionary trajectories of one another, but can also be applied to gene-culture interactions.

## Methods

We consider two cultural traits: **T**, a recipient trait that determines a cultural phenotype, and **N**, a niche constructing trait that determines selection and assortative mating parameters that influence the dynamics of the **T** trait. Each has two possible states (**T**: *T*, *t* and **N**: *N*, *n*), thus there are four possible phenotypes – *TN* (type 1), *Tn* (type 2), *tN* (type 3), and *tn* (type 4), whose population frequencies are denoted by *x*_1_, *x*_2_, *x*_3_, and *x*_4_, respectively, with 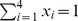. The relative fitnesses of *T* and *t* individuals depend on the state of the **N** trait, as shown in Table 1. Individuals with the *t* trait always have a relative fitness equal to 1, but the relative fitnesses of *TN* and *Tn* can differ. The state of the **N** trait sets the value of the selection coefficient *σ_i_* (−1≤*σ_i_*≤1), such that the phenotype *TN* has fitness 1+*σ*_1_ and the phenotype *Tn* has fitness 1+*σ*_2_.

**Table 1:** Relative fitnesses of the four phenotypes.

Phenotype	Relative fitness
*TN*	1+*σ*_1_
*Tn*	1+*σ*_2_
*tN*	1
*tn*	1

The relative fitness of individuals carrying the *T* trait can differ from that of individuals carrying the *t* trait. The amount of this difference is dictated by the **N** state: the *N* trait confers a fitness difference of *σ*_1_ between *TN* and *tN*, and the *n* trait confers a fitness difference of *σ*_2_ between *Tn* and *tn*.

The state of the **N** trait also determines the value of an assortative mating parameter, which measures the departure from random mating. We define a ‘choosing parent,’ arbitrarily assigned as the father in the subsequent analysis. The choosing parent's **N** state dictates the level of assortative mating, that is, the degree to which an individual of a given **T** state will preferentially mate with another individual of the same state, expressed by parameters *α_i_* (0≤*α_i_*≤1). In the population, a fraction (1−*α_i_*) of individuals will mate randomly, while the remainder of the population (*α_i_*) will mate preferentially with individuals of the same **T** state. If the choosing parent is *N*, individuals mate randomly with probability 1−*α*_1_ and mate preferentially with individuals of the same **T** state with probability *α*_1_, whereas if the choosing parent is *n*, individuals mate randomly with probability 1−*α*_2_ and mate preferentially with individuals of the same **T** state with probability *α*_2_.

There are sixteen father-mother pairs possible from the four phenotypes described here, and we use the notation *m_i,j_* to indicate the frequency of a mating between a father of type *i* and a mother of type *j* where *i*, *j* = {1, 2, 3, 4}; the mating frequency of each pairing is given in Table 2. With preferential mating based on their **T** state, the mating frequency for individuals of different **T** states is the product of the frequency of each phenotype multiplied by the probability of individuals mating at random (1−*α_i_*). The mating frequency for individuals of the same **T** state is the sum of the probability that the individuals mate at random and the probability that the individuals mate assortatively. Since the traits in question are transmitted vertically, for each phenotype we must specify the probability that the mating produces an offspring of that phenotype. These probabilities, *b_i_* and *c_i_* for *i* = {0, 1, 2, 3} shown in Table 3, are assumed to be constant (0≤*b_i_*≤1, 0≤*c_i_*≤1).

**Table 2:** Mating frequencies for all possible matings.

♂×♀	mating frequency	♂×♀	mating frequency
*TN*×*TN*	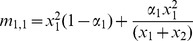	*tN*×*TN*	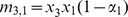
*TN*×*Tn*	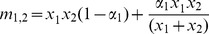	*tN*×*Tn*	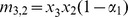
*TN*×*tN*	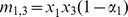	*tN*×*tN*	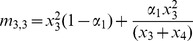
*TN*×*tn*	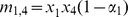	*tN*×*tn*	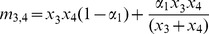
*Tn*×*TN*	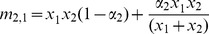	*tn*×*TN*	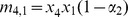
*Tn*×*Tn*	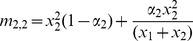	*tn*×*Tn*	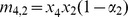
*Tn*×*tN*	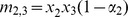	*tn*×*tN*	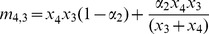
*Tn*×*tn*	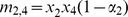	*tn*×*tn*	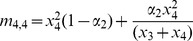

In this model, *α*_1_ is the rate of assortment if the choosing parent is *N*, and *α*_2_ is the rate of assortment if the choosing parent is *n*. The choosing parent is listed first for each mating. On the right side of the equations, the first term represents the frequency of random matings and the second term the frequency of assortative matings.

**Table 3:** Probabilities of offspring outcomes from cultural trait pairings.

	*T*	*t*		*N*	*N*
*T*×*T*	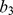	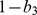	*N*×*N*	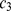	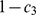
*T*×*t*	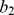	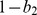	*N*×*n*	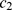	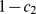
*t*×*T*	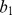	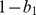	*n*×*N*	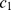	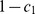
*t*×*t*	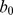	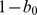	*n*×*n*	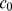	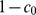

For each mating, the probability of transmitting each trait is given. For example, a mating between a *T* individual and another *T* individual will result in a *T* offspring with probability *b*_3_ and a *t* offspring with probability (1−*b*_3_).

**T** and **N** are assumed to be transmitted independently, so the probability of offspring outcomes for each of the sixteen possible matings is obtained by multiplying the corresponding probabilities from each side of Table 3. For example, a mating of a *TN* individual with a *Tn* individual will produce a *TN* offspring with probability *b*_3_ *c*_2_ and a *Tn* offspring with probability *b*_3_ (1−*c*_2_). If *b*_0_ = 0 and *b*_3_ = 1, then there is no cultural ‘mutation’ from one **T** state to another: two *T* parents will always produce a *T* offspring and two *t* parents will always produce a *t* offspring. In addition, these transmission parameters could take values that represent Mendelian inheritance: *b*_0_ = 0, *b*_1_ = *b*_2_ = 0.5, and *b*_3_ = 1. However, if *b*_0_>0 and *b*_3_<1, there is some rate at which two *T* parents can produce *t* offspring and vice versa. The corresponding statements are true of *c_i_* with respect to the **N** state. This cultural mutation may also be viewed as frequency-independent horizontal transmission.

To compute the frequency of a given phenotype in the next generation, we multiply each mating frequency by the probability that the mating produces that offspring phenotype and sum over each of the sixteen possible mating combinations. Selection, in terms of *σ*_1_ and *σ*_2_, then operates on these offspring. The full recursions, giving 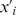, the phenotype frequencies in the next generation, in terms of 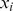 in the current generation, are given in Text S1. If 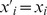, for *i* = {1, 2, 3, 4}, the system is at equilibrium, and the number and structure of these equilibria, as well as whether they are stable, depend on the values of the parameters in Tables 1, 2, 3. We can then combine this analysis of the model with numerical iterations to explore the parameter space (−1≤*σ_i_*≤1, 0≤*α_i_*≤1, 0≤*b_i_*≤1, 0≤*c_i_*≤1) and the nature and stability of the equilibria we find. For a given set of parameter values, we iterate the system until convergence from several initial values of *x_i_* and examine the equilibrium approached from each.

## Results

**Figure 2:**
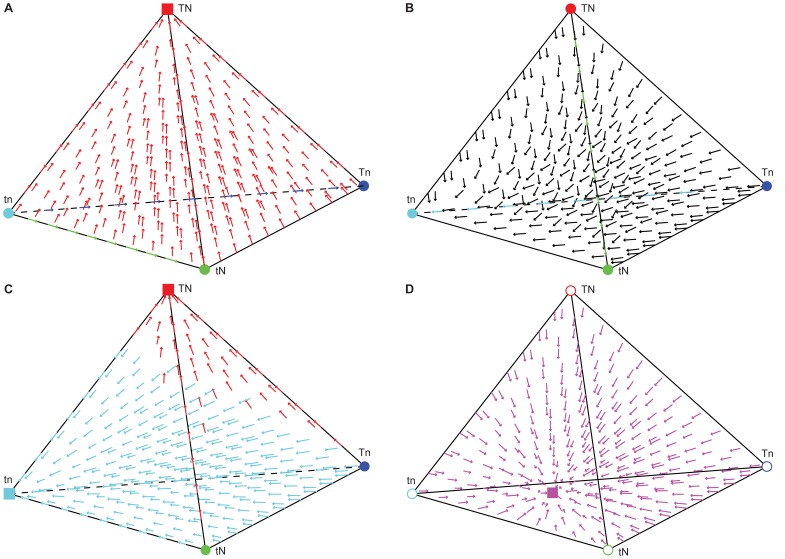
Cultural transmission in different subsets of the parameter space. In this and subsequent figures, a filled square at a vertex indicates a stable fixation at that vertex. A filled circle indicates an equilibrium that is unstable except in a specific hyperplane. Inside the tetrahedron, arrows originate at the population's initial phenotype frequencies and point toward the equilibrium. Arrows are color-coded by the equilibrium approached (*TN*: red, *Tn*: blue, *tN*: green, *tn*: cyan, *tN*-*tn* edge: black, internal polymorphism: pink). **A.** No selection, no assortative mating, no cultural mutation: when *b*_1_+*b*_2_>1 and *c*_1_+*c*_2_>1, the *TN* vertex is stable. When 0<*α*_1_, *α*_2_<1, the same vertex is stable. **B.** No selection, no assortative mating, no cultural mutation: when *b*_1_+*b*_2_<1, the *t* state approaches fixation, and if *c*_1_+*c*_2_ = 1, *N* and *n* persist in their initial proportions. Any point along the edge connecting the *tn* and *tN* vertices can represent an equilibrium. **C.** Selection but no assortative mating, no cultural mutation. For certain parameters, cultural transmission favors fixation of one phenotype but selection favors another. In some of these cases, two fixations are stable and which is approached depends on the initial frequencies. In the case shown here, *α*_1_ = *α*_2_ = 0, *b*_0_ = *c*_0_ = 0, *b*_3_ = *c*_3_ = 1, *b*_1_ = 0.8, *b*_2_ = 0.5, *c*_1_ = 0.5, *c*_2_ = 0.2, *σ*_1_ = −0.2, and *σ*_2_ = −0.6. The transmission favors *T* and *n*, but *Tn* is selected against, so the population approaches fixation of either *TN* or *tn* depending on initial frequencies. **D.** Assortative mating, selection, and cultural mutation. From all initial phenotype frequencies, the population will approach a single stable polymorphism. In this case, *α*_1_ = 0.1, *α*_2_ = 0.1, *b*_0_ = 0.05, *b*_1_ = 0.49, *b*_2_ = 0.52, *b*_3_ = 0.95, *c*_0_ = 0.05, *c*_1_ = 0.51, *c*_2_ = 0.53, *c*_3_ = 0.95, *σ*_1_ = −0.2, and *σ*_2_ = −0.1. At equilibrium, *x*_1_≈0.1438, *x*_2_≈0.0492, *x*_3_≈0.6262, and *x*_4_≈0.1808.

**Figure 3:**
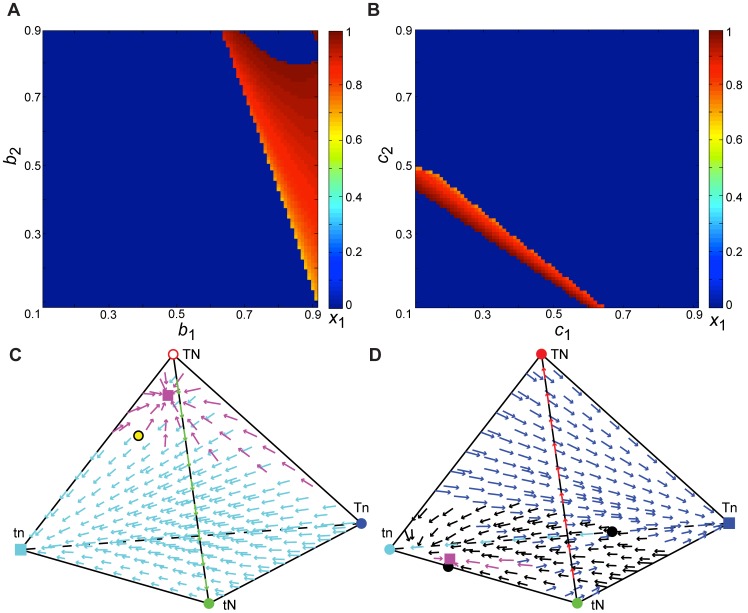
Cultural transmission with assortative mating and selection but no cultural mutation. For most parameter sets, the population approaches a single vertex; in rare cases a stable polymorphism is also present. Panels **A**–**C** show the parameter values *α*_1_ = 0.8, *α*_2_ = 0.3, *b*_0_ = 0, *b*_1_ = 0.7, *b*_2_ = 0.7, *b*_3_ = 1, *c*_0_ = 0, *c*_1_ = 0.5, *c*_2_ = 0.2, *c*_3_ = 1, *σ*_1_ = −0.2, *σ*_2_ = −0.7, and **A**–**B** shows varied pairs of transmission parameters. **A.** The effect of transmission of ***T*** on the presence of a polymorphism. The *x*-axis represents the value of *b*_1_, the *y*-axis represents the value of *b*_2_, and the color scale shows the value of *x*_1_. **B.** The effect of transmission of ***N*** on the presence of a polymorphism. The *x*-axis represents the value of *c*_1_, the *y*-axis represents the value of *c*_2_, and the color scale represents the value of *x*_1_. **C.** The pink square represents a stable polymorphism (*x*_1_≈0.814, *x*_2_≈0.0162, *x*_3_≈0.0937, *x*_4_≈0.0763). Pink arrows illustrate the domain of attraction of this equilibrium. The yellow circle represents an unstable equilibrium between the domains of attraction of the polymorphism and the *tn* vertex. **D.** A polymorphism where *c*_1_+*c*_2_ = 1. For some initial frequencies, the population approaches a single fixed point at the blue square. The pink square represents a stable polymorphic internal equilibrium, pink arrows illustrate the domain of attraction of this equilibrium. Red, green, cyan, and black circles represent unstable equilibria. Black arrows begin at initial conditions that result in an equilibrium on the *tN*-*tn* edge of the tetrahedron. Black circles represent unstable equilibria on the *n* and *t* fixation edges. In this case, *α*_1_ = 0.8, *α*_2_ = 0.3, *b*_0_ = 0, *b*_1_ = 0.2, *b*_2_ = 0.3, *b*_3_ = 1, *c*_0_ = 0, *c*_1_ = 0.3, *c*_2_ = 0.7, *c*_3_ = 1, *σ*_1_ = 0.2, and *σ*_2_ = 0.4.

**Figure 4:**
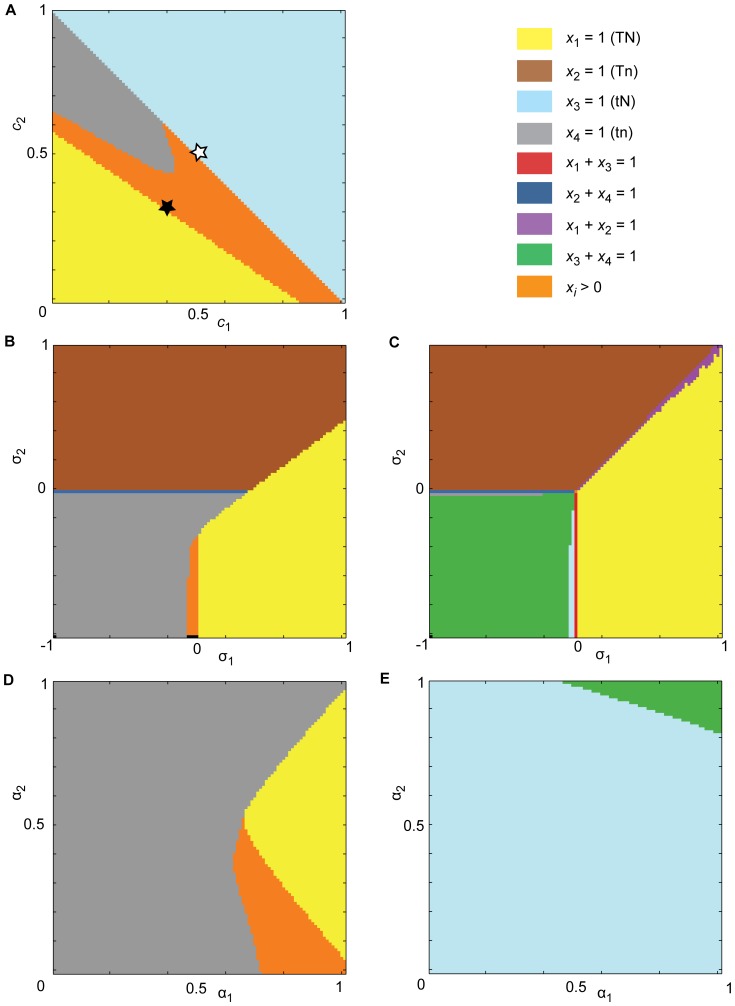
A cultural trait modifying the evolution of a genetic trait. When the **T** trait is transmitted by Mendelian inheritance and the **N** trait is transmitted culturally, assorting and selection may lead to gene-culture polymorphisms. We took the parameter set *α*_1_ = 0.83, *α*_2_ = 0.24, *b*_0_ = 0, *b*_1_ = *b*_2_ = 0.5, *b*_3_ = 1, *c*_0_ = 0, *c*_3_ = 1, *σ*_1_ = −0.01, and *σ*_2_ = −0.82 and varied pairs of parameters as indicated. **A.** Cultural transmission affects equilibria: *c*_1_ and *c*_2_ varied between 0 and 1, and the equilibrium approached from initial frequencies near the *x*_1_−*x*_2_ edge is indicated by color. Polymorphisms exist in the orange region. In **B** and **D**, we considered the transmission parameters indicated by the black star in **A**: *c*_1_ = 0.4 and *c*_2_ = 0.31. In **C** and **E**, we used the Mendelian transmission parameters indicated by the white star in **A**: *c*_1_ = 0.5 and *c*_2_ = 0.5. **B.** Selection parameters that produce a polymorphism are shown in orange. **C.** When both traits show Mendelian transmission, no stable polymorphisms exist for any combination of selection levels. **D.** The assorting parameter combinations that produce a gene-culture polymorphism are shown in orange. **E.** When both traits show Mendelian transmission, polymorphisms do not exist for any combination of assorting parameters.

Three sets of parameters interact in this model: the selection parameters *σ_i_*, assortative mating parameters *α_i_*, and vertical cultural transmission parameters, *b_i_* and *c_i_*. The values of both *σ_i_* and *α_i_* are determined by an individual's **N** state, as described above. In order to study the dynamics of a population with a given set of parameter values, we investigate the possible equilibria, their stability, and the effect of initial phenotype frequencies on the eventual equilibrium reached. Although some special cases are amenable to mathematical solution, most require numerical analysis. For a given set of parameters, we can represent the frequency of each phenotype (*x*_1_, *x*_2_, *x*_3_, and *x*_4_) as a point in the tetrahedron shown in Figures 2, 3, 4, with a vertex representing the fixation of a phenotype; for example, *x*_1_ = 1 at the vertex labeled *TN*. Likewise, a point on the edge between the vertices labeled *tN* and *tn* represents *x*_1_ = *x*_2_ = 0. We include arrows inside the tetrahedron that begin at initial frequencies of each phenotype and point in the direction of the equilibrium approached from these starting frequencies after 50,000 generations.

### Case 1: No selection, no assortative mating no cultural mutation

Here *σ*_1_ = *σ*_2_ = 0 (no selection), *α*_1_ = *α*_2_ = 0 (no assortative mating), *b*_0_ = 0, *b*_3_ = 1 (no cultural mutation of the **T** state), and *c*_0_ = 0, *c*_3_ = 1 (no cultural mutation of the **N** state). The parameters *b*_1_ and *b*_2_ are the probabilities of producing a *T* offspring from a *T*×*t* or a *t*×*T* mating, respectively, and in general these parameters need not be equal. Likewise, *c*_1_ and *c*_2_ correspond to the probability of producing an offspring with an *N* trait from an *N*×*n* or an *n*×*N* mating, respectively. The balance of (*b*_1_+*b*_2_) with (*c*_1_+*c*_2_) dictates the eventual fixation: if *b*_1_+*b*_2_≠1 and *c*_1_+*c*_2_≠1, the system approaches fixation of a single phenotype. For example, if *b*_1_+*b*_2_>1, more offspring with the *T* trait are produced from mixed *T*/*t* matings than offspring with the *t* trait. If *c*_1_+*c*_2_>1 as well, then more *N* offspring are produced from mixed *N*/*n* matings than *n* offspring. If both inequalities hold, *TN* will be favored in the long term, and any initial phenotype frequencies such that 0<*x*_1_, *x*_2_, *x*_3_, *x*_4_<1 will evolve toward *x*_1_ = 1. However, if *N* is initially absent in the population, the population approaches fixation in *Tn* (Figure 2a). If *b*_1_+*b*_2_ = 1 and *c*_1_+*c*_2_ = 1, no phenotype is favored by vertical transmission, and any starting point such that 0<*x*_1_, *x*_2_, *x*_3_, *x*_4_<1 can be an equilibrium. This is referred to as the neutral case.

If *c*_1_+*c*_2_ = 1, which is typical of Mendelian inheritance but is also possible with cultural transmission, then neither *N* nor *n* will be favored and both will be present at equilibrium. For example, if *c*_0_ = 0, *c*_1_ = 0.6, *c*_2_ = 0.4, and *c*_3_ = 1, then if *b*_1_+*b*_2_>1 the *T* state will approach fixation and if *b*_1_+*b*_2_<1 the *t* state will approach fixation, but in both cases *N* and *n* will remain at their original proportions in the population (Figure 2b). In this case, the **N** trait is neutral. Corresponding statements are true for the *T* and *t* states if *b*_1_+*b*_2_ = 1. If both *b*_1_+*b*_2_ = 1 and *c*_1_+*c*_2_ = 1, then both the **T** and the **N** traits are neutral, and *T*, *t*, *N*, and *n* will persist in their initial proportions. Note that while the proportions of these individual states will remain constant, the phenotype frequencies (*x*_1_, *x*_2_, *x*_3_, and *x*_4_) can change from their initial values because offspring can inherit their **T** and **N** traits from different parents.

### Case 2: No selection, assortative mating, no cultural mutation

With *σ*_1_ = *σ*_2_ = 0 (no selection), *α*_1_, *α*_2_>0 (assortative mating), *b*_0_ = *c*_0_ = 0 and *b*_3_ = *c*_3_ = 1 (no cultural mutation), the dynamics are largely similar to those in Case 1 in that the transmission parameters dictate which phenotype ultimately reaches fixation (Figure 2a) unless there is complete assortative mating, in which case the values of the parameters *b*_1_ and *b*_2_ are irrelevant. Thus, when *α*_1_ = *α*_2_ = 1, *c*_1_ and *c*_2_ dictate which of the **N** states will approach fixation and the **T** state is neutral. For example, if *c*_1_+*c*_2_<1, *n* will approach fixation, but any proportions of *Tn* and *tn* can be an equilibrium. Here, the *Tn*-*tn* edge of the tetrahedron is neutrally stable; perturbing the system away from this edge by adding *N* individuals to the population will result in a return to this edge. The proportions of *T* and *t* will not change from generation to generation, but if a perturbation changes these proportions, they will remain at the perturbed frequencies. Likewise, if *c*_1_+*c*_2_>1, the edge between the *TN* and *tN* vertices will be stable when assortative mating is complete.

### Case 3: Selection, no assortative mating, no cultural mutation

Next, we consider the case in which the fitnesses of the phenotypes are not equal, individuals mate randomly, and there is no cultural mutation: −1<*σ*_1_, *σ*_2_<1, *σ*_1_≠*σ*_2_, *α*_1_ = *α*_2_ = 0 (no assortative mating), and *b*_0_ = *c*_0_ = 0 and *b*_3_ = *c*_3_ = 1 (no cultural mutation). In this case, a single phenotype often approaches fixation. However, when vertical transmission favors one phenotype but selection favors another, two vertices may be locally stable, in which case the initial phenotype frequencies dictate which vertex will eventually be approached (Figure 2c).

### Case 4: Selection, assortative mating, cultural mutation

When there is cultural mutation in the population (−1<*σ_i_*<1, 0<*α_i_*, *b_i_*, *c_i_*<1), no boundary can be reached from any starting point. In all cases examined, only one stable polymorphism exists in the interior of the tetrahedron (Figure 2d).

### Case 5: Selection, assortative mating, no cultural mutation

With both selection and assortative mating (−1<*σ*_1_, *σ*_2_<1, *α*_1_, *α*_2_>0) but no cultural mutation (*b*_0_ = *c*_0_ = 0, *b*_3_ = *c*_3_ = 1), stable equilibria with one or both traits fixed are possible. In most such cases, a single phenotype is favored and ultimately approaches fixation, except in populations that are initially missing one of these traits. We tested approximately 25 million combinations of parameters, avoiding values close to zero or one (0.2<*α*_1_, *α*_2_<0.8, 0.2<*b*_1_, *b*_2_<0.8, 0.2<*c*_1_, *c*_2_<0.8, −0.8<*σ*_1_, *σ*_2_<0.8) and found that in a small fraction of cases (on the order of 1 in 50,000), multiple stable equilibria are possible, including one vertex and one polymorphism with all phenotypes at a frequency greater than 0.01, as well as at least one unstable equilibrium. Using these rare polymorphisms as starting points, we could identify patterns of parameter values that allowed for the persistence of all four phenotypes. As an illustration, we consider the case where *α*_1_ = 0.8, *α*_2_ = 0.3, *b*_0_ = 0, *b*_1_ = 0.7, *b*_2_ = 0.7, *b*_3_ = 1, *c*_0_ = 0, *c*_1_ = 0.5, *c*_2_ = 0.2, *c*_3_ = 1, *σ*_1_ = −0.2, and *σ*_2_ = −0.7. If we test numerous combinations of *b*_1_ and *b*_2_ but hold the other parameters constant, we find that a subset of these combinations produce a stable polymorphism and the remainder give fixation of a single phenotype (Figure 3a); likewise, a subset of *c*_1_ and *c*_2_ pairs will result in the stable persistence of all four phenotypes (Figure 3b).

In cases where multiple stable equilibria exist, the equilibrium approached depends on the population's initial composition. For example, with the set of parameters listed above, an interior stable polymorphism exists, and from outside of its domain of attraction the population approaches fixation of one phenotype (Figure 3c). Which phenotype approaches fixation depends on the relationship between the parameters. For example, if *α*_2_>*α*_1_, *c*_1_+*c*_2_>1, *σ*_2_>*σ*_1_, *σ*_1_<0, and *σ*_2_<0, then *x*_3_ = 1 tends to be locally stable in addition to the stable polymorphism. Similarly, when *α*_1_>*α*_2_, *c*_1_+*c*_2_<1, and *σ*_1_>*σ*_2_, *x*_4_ = 1 is likely to be stable in addition to the stable polymorphism. In both of these situations there is one unstable fixation and another unstable equilibrium between the polymorphism's domain of attraction and the stable fixation point.

In certain cases, parameter combinations can produce quite complex outcomes, especially when the cultural transmission parameters from mixed matings sum to one for one trait: from certain initial frequencies a stable interior polymorphism is approached, whereas from other initial frequencies, fixation in one phenotype is approached, while other starting points are neutral with respect to one of the traits (an edge of the tetrahedron). In Figure 3d, for example, with *α*_1_ = 0.8, *α*_2_ = 0.3, *b*_0_ = 0, *b*_1_ = 0.2, *b*_2_ = 0.3, *b*_3_ = 1, *c*_0_ = 0, *c*_1_ = 0.3, *c*_2_ = 0.7, *c*_3_ = 1, *σ*_1_ = 0.2, and *σ*_2_ = 0.4, *Tn* fixation (*x*_2_ = 1) is locally stable, and there is a stable polymorphism with all four phenotypes present (*x*_1_≈0.0176, *x*_2_≈0.0284, *x*_3_≈0.2558, and *x*_4_≈0.6981). In addition, there are four unstable equilibria: two distinct fixation points (*TN* can approach fixation when *n* is completely absent and *tn* can approach fixation when *N* is completely absent and *x*_2_<0.643), one point between the domains of attraction of the stable polymorphism and the neutral edge, and one point between the domains of attraction of the *Tn* vertex and the *tn* vertex. Further, the domain of attraction of the neutral edge does not include all initial phenotype frequencies near it. If the initial conditions are close to fixation in *t*, that is, *x*_1_+*x*_2_≪*x*_3_+*x*_4_ but all *x_i_*>0, the system will approach different equilibria depending on the initial proportions of *N* and *n* in the population. For example, with the parameters above, if *x*_3_>0.735 initially, the population will approach fixation at *x*_2_ = 1, but if *x*_3_<0.735 initially, the population will approach an equilibrium in which *x*_3_ and *x*_4_ are both present. For most initial frequencies with *x*_3_<0.735, *x*_3_+*x*_4_ = 1 at equilibrium, but there is a set of initial conditions near the *tN*-*tn* edge, where 0.261<*x*_3_<0.372 and *x*_1_ and *x*_2_ are close to zero, that lead to an equilibrium with all four phenotypes present. This example illustrates that a single set of parameters for selection, assortative mating, and cultural transmission can result in a diverse set of evolutionary outcomes depending on the founding history of the population.

### Case 6: Gene-culture coevolution

Finally, we consider the case in which individuals can mate assortatively and the fitnesses of the phenotypes are not equal (−1<*σ*_1_, *σ*_2_<1, *σ*_1_≠*σ*_2_, *α*_1_, *α*_2_>0) but one of the traits follows Mendelian transmission rules. Thus, a culturally transmitted trait is modifying the evolution of a genetically inherited trait or vice versa. In this case, the genetically transmitted trait often approaches fixation, and the culturally transmitted trait tends to approach fixation or an equilibrium between the two cultural phenotypes. However, with certain levels of selection and assorting, a culturally inherited trait (**N**) can modify the evolutionary dynamics of a genetic trait (**T**), resulting in the stable persistence of all four phenotypes. Likewise, a genetically inherited trait (**N**) can modify the evolution of a cultural trait (**T**) to produce a polymorphism. These polymorphisms can be found in cases with and without cultural mutation of the culturally transmitted trait. In contrast, if both traits exhibit Mendelian inheritance, no combinations of assorting and selection appear to result in a polymorphism where all four genotypes are present in the population: at least one set of non-Mendelian transmission parameters seems to be necessary for a polymorphic equilibrium. By varying the transmission, selection, and assorting parameters in turn while maintaining Mendelian inheritance of one trait, we find regions of the parameter space that result in the persistence of all four phenotypes, but only when the transmission of the other trait is non-Mendelian (Figure 4).

## Discussion

The term ‘cultural niche construction’ encompasses two types of cultural processes [4]. In culture-culture interactions, a cultural trait changes the selection pressures on, or the transmissibility of, other cultural traits. The other is a process generating feedback between cultural evolution and genetic evolution leading to gene-culture coevolution [6]. The model presented above can represent either of these processes depending on the choice of transmission parameters. The feedback in the model is generated through the interaction of the selection parameters *σ_i_*, the assorting rates *α_i_*, and the transmission rates *b_i_* and *c_i_*. The **T** and **N** traits can be culturally transmitted, and **N** affects the relative fitnesses of *T* and *t* (see Table 1). The **N** trait thus influences the evolution of the population as a result of its culturally induced effect on the **T** trait. This is cultural niche construction, the strength and characteristics of which depend on all three sets of parameters in our model: the transmission rates, the selection pressures, and the levels of assortative mating.

Our model can represent gene-culture coevolution in either of two contexts: a genetically inherited trait that modifies the evolution of a culturally inherited trait, and vice versa. When one of the two traits exhibits Mendelian inheritance (for example, *b*_0_ = 0, *b*_1_ = *b*_2_ = 0.5, *b*_3_ = 1) and the other is not Mendelian, most combinations of cultural transmission, selection, an assorting lead to equilibria in which the genetically inherited trait is fixed. However, as with two culturally transmitted traits (Case 5), the transmission, assorting, and selection can be balanced in such a way as to result in stable polymorphisms of all four phenotypes. Either case of gene-culture coevolution may result in polymorphisms if the cultural transmission, selection, and assorting interact appropriately. With this model, we observed polymorphisms both when cultural mutation is present and when it is absent. However, no combination of assorting and selection parameters was found to give stable polymorphisms when both **T** and **N** were inherited according to Mendelian rules in this model. This underscores the evolutionary importance of the interaction between cultural transmission, selection, and assorting. Our model may be applied to a wide range of cultural niche construction systems, including three often studied social applications: the cultural evolution of religion and high fertility, the cultural evolution of war, and the cultural evolution of sex ratio bias, which is strong in several parts of the world and can interact with mating customs [25].

### Cultural evolution of religion and fertility

The cultural evolution of religious belief and its effects on in- and out-group acceptance and conflict have been widely studied, and attempts have been made to explain the evolution of both the human capacity for religious acceptance and its persistence as a cultural belief [22], [26], [27]. The interaction between religiosity and fertility discussed by Rowthorn [22] can also be described by our model, although there are some fundamental differences between his model and ours. After Rowthorn [22], we can suppose that one of our traits controls a genetic predisposition to religiosity (**N**) and the other determines the cultural belief in religion (**T**). We follow Rowthorn's assumption that there is complete assortative mating according to religious belief, **T**, (*α*_1_ = *α*_2_ = 1). The **N** trait is transmitted genetically, that is, *c*_0_ = 0, *c*_3_ = 1, and *c*_1_ = *c*_2_ = 0.5. The complete assortative mating renders the parameters *b*_1_ and *b*_2_ irrelevant since a *T* individual will not mate with a *t* individual. In his model, Rowthorn [22] includes parameters controlling what he describes as ‘switching;’ these are the probabilities that an individual adopts the opposite state of the cultural trait from the phenogenotype inherited through vertical transmission. In his model, there are four such switching parameters, one for each phenotype. Switching from non-belief (n) to religious belief (r) is considered more likely for an individual possessing the religiosity allele (R) than the non-religiosity allele (N), and, likewise, switching to non-belief is more likely for an individual with the non-religiosity allele. Rowthorn assumes 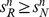 (where 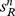 represents the probability that an individual of phenotype *nR* will switch to *rR*, and so on) and 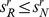; in other words, the religiosity gene predisposes individuals to religious belief because the probability of switching to religious belief, from *n* to *r*, is greater for individuals carrying *R* and vice versa for carriers of *N*. The transmission here does not involve conversion by contact with individuals of another type (horizontal transmission as defined by Cavalli-Sforza and Feldman [28]), but occurs at a constant rate for each phenotype: it is not frequency dependent and can be viewed as mutation rather than cultural transmission.

Rowthorn's condition 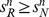 and 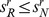 cannot be matched exactly in our model, where transmission of **T** is independent of transmission of **N**, so the probability of cultural mutation depends on the frequencies of the relevant states, which can change over time and with different initial frequencies. For example, the frequency of a cultural mutation from *T* to *t* is the total frequency of *T*×*T* matings in the population (when *α*_1_ = *α*_2_ = 1, *TN*×*TN* matings occur with frequency 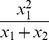, *TN*×*Tn* and *Tn*×*TN* both with frequency 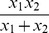, and *Tn*×*Tn* with frequency 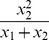, following Table 2) multiplied by the probability of producing a *t* offspring from a *T*×*T* mating, 1−*b*_3_. Thus, the actual rate of *T* to *t* mutation can be viewed as 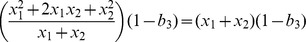, which is not affected by the **N** phenotype. Rowthorn's model predicts that, regardless of the strength of selection in favor of the ‘religious predisposition’ allele, and even with high defection from religious to non-religious sects, the religiosity allele will eventually fix if fertility is higher in the religious groups and the switching rates follow the inequalities listed above (Table 1, [22]). With cultural mutation (*b*_0_>0, *b*_3_<1), as well as the conditions outlined above (*α*_1_ = *α*_2_ = 1, *c*_0_ = 0, *c*_3_ = 1, *c*_1_ = *c*_2_ = 0.5, *σ*_1_, *σ*_2_>0, *σ*_1_≠*σ*_2_), there are two potential equilibrium points, one on the *TN*-*tN* edge and one on the *Tn*-*tn* edge, and which of these equilibria is approached depends on the relationship between the selection parameters. If *σ*_1_>*σ*_2_>0, such that fertility is higher in religious groups (*T* is favored over *t*) and those individuals with the religiosity allele are more likely to become religious (*TN* is favored over *Tn*), we reach the same conclusions as Rowthorn [22]: the genetically transmitted religiosity allele approaches fixation and the culturally transmitted religious belief approaches an equilibrium determined by the transmission parameters *b*_0_ and *b*_3_. In the tetrahedron, there is a single stable equilibrium on the edge between the *TN* (*r, R*) vertex and the *tN* (*n, R*) vertex, corresponding to fixation of the religiosity allele and persistence of both cultural states (religious belief and non-belief), as observed by Rowthorn [22]. The rate of cultural mutation (*b*_0_ and *b*_3_) determines the ratio of believers to non-believers at equilibrium. Given these assumptions, however, our result is similar to Rowthorn's but does not rely on the religiosity trait conferring a predisposition to religion since the genetic trait here does not impose directionally biased mutation of the cultural trait according to phenotype-specific switching rates. Instead, the genetic trait is much less specific, producing a differential selective advantage to one cultural trait over another. Indeed, if conversion occurs between the cultural states of religious belief and non-belief, then continued presence of both belief and non-belief is inevitable because neither state can reach stable fixation. Rowthorn presents an interesting model to explain the persistence of both religious belief and non-belief in humans as an alternative to an evolutionary ‘spandrel’ theory [29]. Our model gives similar results without the constraint that religious predisposition is genetic, as long as cultural mutation is possible and there is a fitness advantage to the cultural trait in question.

However, Rowthorn [22] makes a series of important assumptions that may affect the outcomes of his model. The most striking of these is complete assortment among members of religious (and non-religious) groups. Rowthorn further assumes that religious individuals (*T*), regardless of their genetic background, demonstrate a certain level of increased fertility. This regime of assorting and selection in our model, namely *α*_1_ = *α*_2_ = 1 and *σ*_1_ = *σ*_2_>0, results in a selectively neutral line of possible polymorphisms connecting the *TN*-*tN* edge to the *Tn*-*tn* edge. The exact polymorphism approached depends on the starting conditions.

**Figure 5:**
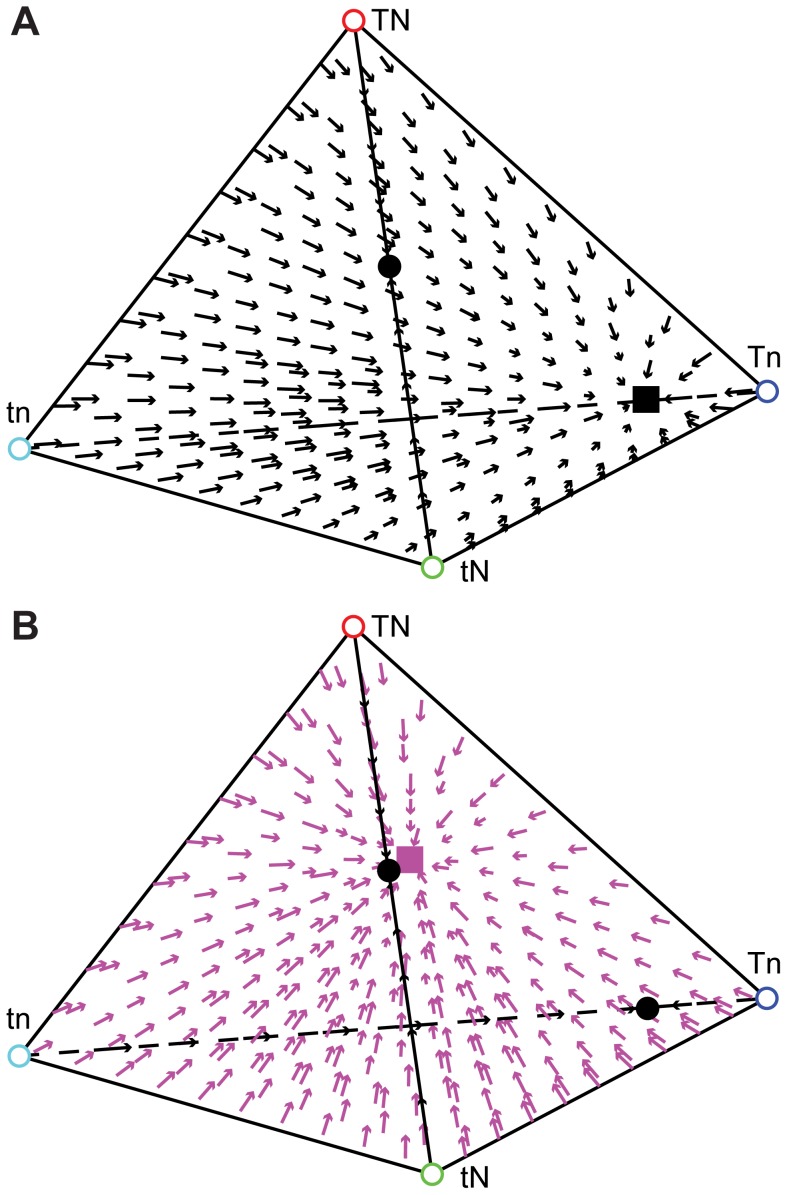
Model for the evolution of religious beliefs. Small fitness differences can alter the evolutionary dynamics of cultural traits. **N** represents the genetically transmitted religious predisposition trait and **T** represents the culturally transmitted belief trait. For both panels, *α*_1_ = 0.73, *α*_2_ = 0.94, *b*_0_ = 0.02, *b*_1_ = 0.3, *b*_2_ = 0.31, *b*_3_ = 0.98, *c*_0_ = 0, *c*_1_ = *c*_2_ = 0.5, and *c*_3_ = 1. **A.** When *σ*_1_ = *σ*_2_ = 0.12, a stable equilibrium exists on the *Tn*-*tn* edge (black square), i.e. fixation of the non-religiosity allele (*n*) and a polymorphism between religious belief (*T*) and non-belief (*t*), which is approached from all starting points except those on the *TN*-*tN* edge, which approach the equilibrium illustrated by the black circle. **B.** When *σ*_1_ = 0.12 and *σ*_2_ = 0.11, a stable polymorphism (pink square) exists such that both religious and non-religious predispositions, as well as religious belief and non-belief, coexist in the population (*x*_1_≈0.521, *x*_2_≈0.127, *x*_3_≈0.295, *x*_4_≈0.057). This polymorphism is approached from all starting points except those on the *TN*-*tN* or *Tn*-*tn* edges, which approach the equilibrium illustrated by the black circles.

Many religious groups have high rates of endogamy, as noted by Rowthorn, but religious groups are unlikely to have *perfect* endogamy and some mixing is inevitable [30]. Relaxing Rowthorn's assumption, we allow assorting to be high but not complete. Data in [30] suggest that rates of endogamy within the religious groups surveyed were between 0.618 and 0.914 at the time of survey. Expanding this range slightly, we investigate the outcomes of our model for the range 0.6<*α*_1_, *α*_2_<1. This enables us to take account of the important effects of mixed marriages in the evolution of religiosity. In this case, we find a number of polymorphisms dependent on the values of the assortative mating parameters, the cultural transmission of religious beliefs to children of mixed marriages, and the selection pressures. Small differences in the selection pressures, however, can lead to fixation of the genetic trait while both states of the cultural belief trait persist (Figure 5). Although Rowthorn makes a series of suggestions regarding possible situations in which the religiosity allele may not be driven to fixation (heterozygote advantage, convergence of religious and non-religious birth rates etc.), he does not consider the effect of relaxing his strong assumption of complete assorting. We show here that stable polymorphisms are possible if we allow for the possibility of a small number of mixed marriages.

### Cultural evolution of large-scale conflict

Our model may also help to understand in- and out-group interactions that contribute to conflict and how conflict might be alleviated. Hinde [31] suggests that it is the culturally driven exploitation of genetic predispositions towards self-defense that leads to modern large-scale conflicts. The spread of violent tendencies in society could be largely facilitated by horizontal transmission ‘catalyzed by predispositions…that leave individuals particularly receptive to propaganda messages’ [32]. In terms of our model, the cultural mutation parameters *b*_0_, *c*_0_, 1−*b*_3_, and 1−*c*_3_ become very important in determining the eventual frequencies of cultural traits in the population. Since horizontal transmission is not included we can interpret these cultural mutations as representing any factor that changes the beliefs of offspring relative to those of their parents. Consider the investigation by Halperin, et al. [23] of the Israeli-Palestinian conflict. They exposed Israeli Jews, Palestinian citizens of Israel, and Palestinians in the West Bank to reading material suggesting that groups in general were either malleable in their beliefs or, alternatively, that they were fixed and unchanging in their beliefs. All of the subject groups responded to material suggesting groups were malleable with an ‘increased willingness to compromise for peace’ [23]. This type of culture-culture interaction can also be modeled using our system; we can characterize one cultural dichotomy as the willingness to compromise for peace (*T*/*t*), and the other as a cultural modifier, namely an individual's belief in the malleability of groups (*N*/*n*). Individuals who place a high value on compromise might be more likely to partner with other compromisers, and, likewise, those unwilling to compromise for peace might preferentially associate with those who are also uncompromising. This entails assortative mating (or, more likely, assortative meeting, as in [33]) based on the state of an individual's **T** trait.

Although the selection acting on such complex cultural traits is difficult to characterize, we can make some simplifying assumptions. Lehmann and Feldman [34] describe a model of ‘belligerence and bravery,’ two conflict-related traits. Belligerence increases the likelihood of aggression and bravery increases the likelihood of victory in the conflicts initiated by acts of aggression. The selection pressures acting on those individuals who engage in war-like behaviors are complex. On the one hand, they may have a shorter lifespan than their more peaceful counterparts, but the increased gain of fitness-enhancing resources may balance this loss. However, Lehmann and Feldman's model is probably most relevant to tribal warfare where space and access to resources and mates could be important factors in interactions with out-groups. In many modern conflicts this may no longer be the case, because the motivations and goals of large-scale industrial societal conflicts are far from fitness maximization of the individuals who actually fight [35] and depend on factors at the level of the whole society [31]. We might assume that the evolutionary effect of reduced life expectancy for present-day combatants far outweighs any benefits accrued from increased access to resources and mates in conquered land. Thus, in applying our model to modern conflicts, we might suppose that selection favors compromising traits (*T*) over cultural beliefs that favor war (*t*). However, the societal pressures (e.g. manipulative media or ‘mobilizing and abusive leaders’ [36]) may cause the offspring of ‘compromisers’ to change beliefs (or actions), thus maintaining war-like phenotypes.

Such a model applied to modern warfare, therefore, is analogous to Case 4 described above with *σ*_1_, *σ*_2_>0, *α*_1_, *α*_2_>0, and 0<*b_i_*, *c_i_*<1, where we see that, from a system initially containing all cultural traits (*TN, Tn, tN, tn*), an equilibrium in which one phenotype fixes is impossible and there is just one polymorphic equilibrium, which is critically dependent on the mutation parameters and the level of assortative mating. The model raises an interesting possibility: to the extent that a belief in group malleability is correlated with a belief in individual malleability, it may be the case that individuals lacking belief in the ability of groups (and hence individuals) to change (*n*) might choose to associate with others that share their beliefs about compromising (**T**), while those who do believe in group and individual malleability (*N*) might not preferentially partner with others that already share their beliefs, corresponding to a high value for *α*_2_ and a low value for *α*_1_. This could in turn lead to a population-level increase in the willingness to compromise for peace over populations in which believers in malleability also choose to assort preferentially, provided that the relative ability of *T* individuals to spread their beliefs to the next generation in mixed marriages is high enough.

### Cultural evolution of sex ratio

Our model can also be applied to the cultural evolution of sex ratio bias. In China, over the past thirty years decreasing total fertility has been correlated with increasing male bias in sex ratio at birth, leading to an increasing excess of males, which has the potential for dramatic societal ramifications [21], [37], [38] as well as consequences for the primary sex ratio [39], [40]. In addition to the ethical concerns about sex-selective abortion and infanticide, marriage prospects for males, especially poor rural males, continue to deteriorate as the children born after the institution of China's family planning policies reach marrying age. In applying our model, we can consider **T** to be a son preference trait and **N** to be a cultural modifier of this trait. An individual with *T* exhibits son preference, and an individual with *t* has no preference. The *N* and *n* states might modulate the degree to which individuals will take their partner's son preference into account when choosing a mate (i.e. assortative mating based on son preference) and the fitness benefit or cost conferred upon those who exhibit son preference (i.e. selection). It is not unrealistic to assume that an individual might not demonstrate exactly the same cultural beliefs (*T* or *t*) as his or her parents in this context; two parents with the same state might produce an offspring with the other state. As shown in Case 4, when this kind of cultural mutation is permitted, the equilibrium always has all four types present, and the location of this polymorphism depends on the exact parameter values. If we consider a scenario in which people are more likely to marry an individual who shares their cultural beliefs (*α*_1_, *α*_2_>0), then sons are less likely to find a mate than daughters and fitness is decreased for those that practice son preference (*σ*_1_, *σ*_2_<0). This would produce an equilibrium with more individuals exhibiting no son preference (Figure 2d), and we can test the relative importance of selection, assortative mating, and cultural transmission in determining the equilibrium frequency of son preference. An alternative framework would have the *N*/*n* dichotomy determine a preference for virilocal marriage, in which a wife moves to her husband's natal home after marriage, or no such preference. There is some evidence that virilocal marriage is correlated with an increase in the likelihood of sex selection of a fetus [25], which is the behavioral expression of son preference.

Our model of cultural evolution provides a framework for investigating the evolution of a diverse set of interacting human behaviors. We can explore cases of cultural niche construction in which one cultural trait alters the selective environment of another cultural trait, gene-culture coevolution in which a cultural trait changes selection pressures on a genetic trait, and situations in which a genetic trait influences the selection pressures on a cultural trait. The evolutionary dynamics depend on the balance between the parameters regulating cultural transmission, selection, and assortative mating. We considered neutral values for each of these sets of parameters in turn and observed that polymorphisms can only persist when both assortative mating and selection are included and at least one trait exhibits non-Mendelian inheritance, unless cultural mutation makes such polymorphisms inevitable. Although we have suggested a few areas where the framework of our model could be applicable, many more applications of this kind of cultural niche construction may be possible.

## Supporting Information

Text S1
**Recursions.** Equations A1–A4 describe the relationship between the phenotype frequencies in the current generation, *x_i_*, and those in the next generation, 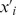. The average fitness (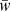) is the sum of the right side of these four equations and acts to normalize 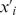 so that 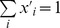.(DOC)
