# Supplementary material for: Models of Cultural Niche Construction with Selection and Assortative Mating
Source: PLoS One. 2012 Aug 14;7(8):e42744. doi: 10.1371/journal.pone.0042744 (PMC3419226; doi:10.1371/journal.pone.0042744)
Supplement: Text S1 — Recursions. Equations A1–A4 describe the relationship between the phenotype frequencies in the current generation, xi, and those in the next generation, . The average fitness () is the sum of the right side of these four equations and acts to normalize so that . (DOC) [file pone.0042744.s001.doc]

**Text S1: Recursions**

$$\begin{matrix} \bar{w}x'_{1}=(1+\sigma_{1})(m_{1,1}b_{3}c_{3}+m_{1,2}b_{3}c_{2}+m_{1,3}b_{2}c_{3}+m_{1,4}b_{2}c_{2}+m_{2,1}b_{3}c_{1}+m_{2,2}b_{3}c_{0}+m_{2,3}b_{2}c_{1}+ \\ m_{2,4}b_{2}c_{0}+m_{3,1}b_{1}c_{3}+m_{3,2}b_{1}c_{2}+m_{3,3}b_{0}c_{3}+m_{3,4}b_{0}c_{2}+m_{4,1}b_{1}c_{1}+m_{4,2}b_{1}c_{0}+m_{4,3}b_{0}c_{1}+m_{4,4}b_{0}c_{0}) \end{matrix}$$

(A1)

$$\begin{matrix} \bar{w}x'_{2}=(1+\sigma_{2})(m_{1,1}b_{3}(1-c_{3})+m_{1,2}b_{3}(1-c_{2})+m_{1,3}b_{2}(1-c_{3})+m_{1,4}b_{2}(1-c_{2})+ \\ m_{2,1}b_{3}(1-c_{1})+m_{2,2}b_{3}(1-c_{0})+m_{2,3}b_{2}(1-c_{1})+m_{2,4}b_{2}(1-c_{0})+ \\ m_{3,1}b_{1}(1-c_{3})+m_{3,2}b_{1}(1-c_{2})+m_{3,3}b_{0}(1-c_{3})+m_{3,4}b_{0}(1-c_{2})+ \\ m_{4,1}b_{1}(1-c_{1})+m_{4,2}b_{1}(1-c_{0})+m_{4,3}b_{0}(1-c_{1})+m_{4,4}b_{0}(1-c_{0})) \end{matrix}$$

(A2)

$$\begin{matrix} \bar{w}x'_{3}=m_{1,1}(1-b_{3})c_{3}+m_{1,2}(1-b_{3})c_{2}+m_{1,3}(1-b_{2})c_{3}+m_{1,4}(1-b_{2})c_{2}+ \\ m_{2,1}(1-b_{3})c_{1}+m_{2,2}(1-b_{3})c_{0}+m_{2,3}(1-b_{2})c_{1}+m_{2,4}(1-b_{2})c_{0}+ \\ m_{3,1}(1-b_{1})c_{3}+m_{3,2}(1-b_{1})c_{2}+m_{3,3}(1-b_{0})c_{3}+m_{3,4}(1-b_{0})c_{2}+ \\ m_{4,1}(1-b_{1})c_{1}+m_{4,2}(1-b_{1})c_{0}+m_{4,3}(1-b_{0})c_{1}+m_{4,4}(1-b_{0})c_{0} \end{matrix}$$

(A3)

$$\begin{matrix} \bar{w}x'_{4}=m_{1,1}(1-b_{3})(1-c_{3})+m_{1,2}(1-b_{3})(1-c_{2})+m_{1,3}(1-b_{2})(1-c_{3})+m_{1,4}(1-b_{2})(1-c_{2})+ \\ m_{2,1}(1-b_{3})c_{1}+m_{2,2}(1-b_{3})c_{0}+m_{2,3}(1-b_{2})c_{1}+m_{2,4}(1-b_{2})c_{0}+ \\ m_{3,1}(1-b_{1})(1-c_{3})+m_{3,2}(1-b_{1})(1-c_{2})+m_{3,3}(1-b_{0})(1-c_{3})+m_{3,4}(1-b_{0})(1-c_{2})+ \\ m_{4,1}(1-b_{1})(1-c_{1})+m_{4,2}(1-b_{1})(1-c_{0})+m_{4,3}(1-b_{0})(1-c_{1})+m_{4,4}(1-b_{0})(1-c_{0}) \end{matrix}$$

(A4)
